# Green Synthesis of Biogenic Silver Nanoparticles for Efficient Catalytic Removal of Harmful Organic Dyes

**DOI:** 10.3390/nano10020202

**Published:** 2020-01-24

**Authors:** Luminita David, Bianca Moldovan

**Affiliations:** Research Center for Advanced Chemical Analysis, Instrumentation and Chemometrics (ANALYTICA), Faculty of Chemistry and Chemical Engineering, Babes-Bolyai University, 400028 Cluj-Napoca, Romania; muntean@chem.ubbcluj.ro

**Keywords:** green synthesis, silver nanoparticles, *Viburnum opulus* L., tartrazine, carmoisine, brilliant blue FCF

## Abstract

The present article reports an environmentally benign method for synthesizing silver nanoparticles using the fruit extract of *Viburnum opulus* L. as a source of bioactive compounds, which can act as reducing agents of the silver ions and also as stabilizing agents of the obtained nanoparticles. The catalytic ability of the synthesized silver nanoparticles (AgNPs) to remove toxic organic dyes was also evaluated. The biosynthesis of silver nanoparticles was firstly confirmed by UV-Vis spectral analysis, which revealed the presence of the characteristic absorption peak at 415 nm corresponding to the surface plasmon vibration of colloidal silver. Fourier-transform infrared spectroscopy (FTIR) and thermogravimetric analysis (TGA) studies were conducted to confirm the presence of bioactive phytocompounds, especially phenolics, as capping and stabilizing agents of the AgNPs. The size, morphology and crystalline nature of the synthesized AgNPs were investigated by transmission electron microscopy and X-ray diffraction techniques revealing that the obtained nanoparticles were spherical shaped, with an average diameter of 16 nm, monodispersed, face centered cubic nanoparticles. Further, the catalytic ability in the degradation of tartrazine, carmoisine and brilliant blue FCF dyes by NaBH_4_ was evaluated. The results demonstrated an efficient activity against all the investigated dyes being an outstanding catalyst for the degradation of brilliant blue FCF. This eco-friendly synthetic approach can generate new tools useful in environmental pollution control.

## 1. Introduction

The field of nanotechnology has gained lately a tremendous development especially due to the remarkable properties of the nanoscale materials, which significantly differ from those of bulk material. These impressive properties led to various applications in many fields of science such as biomedicine, pharmaceutics, cosmetics, catalysis, textiles and optics [1,2,3,4]. Among metal nanoparticles, silver and gold nanoparticles are widely used because of their applications. The synthesis of nanoscale silver can be easily achieved by various methods, such as physical, photochemical, electrochemical and many others. Most of the synthetic approaches used to convert silver ions into elemental silver involve the use of hazardous chemicals as reducing agents such as hydrazine, sodium borohydride, sodium sulfide, the use of surfactants, polymers and dendrimers as capping agents or imply severe reaction conditions and might generate toxic byproducts, leading to environmental pollution. This negative impact of the above mentioned synthetic methods led to development of new green, eco-friendly synthetic approaches, which use biological resources both as reducing and capping agents of the synthesized nanoparticles. The green synthesis route of noble metal nanoparticles employs natural bioagents like plants [5,6,7], algae and microorganisms such as fungi, bacteria and viruses [8,9,10]. Obtaining nanoparticles by biological means has the advantage of being an eco-friendly, one step, low cost, energy efficient and easy scaling up method. Phytochemicals like polyphenolic compounds, amino acids and proteins, vitamins, polysaccharides and terpenoids play an important role in the green synthesis of silver nanoparticles acting as reducing and stabilizing agents [11,12]. The present article reports the synthesis of silver nanoparticles mediated by the bioactive molecules from *Viburnum opulus* L. fruit extract. *Viburnum opulus* L., known as snowball tree or European cranberry bush, is an endemic plant belonging to the Caprifoliaceae family, wide spread in north-eastern, Central and Eastern Europe. Its fruits are consumed fresh, in beverages and food preparations such as sauces, marmalades and jellies. They are reported to possess a high anti-oxidative effect, especially due to their high amount of polyphenolics, anthocyanins and ascorbic acid [13,14,15,16]. The bioactive phytocomponents of European cranberry bush fruits were also reported to possess antimicrobial, antidiabetic, antihypertensive and anti-inflammatory effects [17,18,19]. The valuable antioxidant compounds of these fruits were already successfully exploited by our research group, to achieve the green synthesis of silver nanoparticles and their in vitro and in vivo anti-inflammatory activity was reported [17].

The non-biodegradable organic dyes resulting from textile, plastic, paper and food industries are usually released in water without any previous treatment, leading to significant environmental pollution. Many of these dyes can cause substantially damages to the aquatic organisms and they are also highly toxic to human life, possessing mutagenic and carcinogenic effects [20,21]. Therefore, dye effluents should be treated to remove these compounds from waste waters. Various techniques such as absorption, chemical, photochemical and biodegradative methods are conventionally applied for this purpose [22]. Dye pollutants are rather resistant to these physical–chemical methods due to their high chemical stability. The removal of these toxic pollutants from these wastewaters is a complex concern and, to this end, a variety of treatments has been designed and developed such as physico-chemical and biological methods, as international environmental safety regulations are becoming more and more stringent [23,24,25]. Efforts are made to find an ideal dye removal method able to efficiently remove a large amount of dye in a short time, without generating further pollution by producing more hazardous by-products. To this end, the using of green synthesized metallic nanoparticles as efficient catalysts for the reduction of organic dyes has been largely explored [26,27,28]. The unique physical, chemical and electronic properties of metal nanoparticles recommend these nanomaterials as good catalysts, suitable for the reductive degradation of organic dyes, being an efficient alternative to conventional methods used for the removal of dye contaminants. Currently, green synthesized silver nanoparticles are intensively used to reduce the dye stuffs from aqueous medium [29,30,31].

The present work aimed to exploit the presence of the antioxidant potential of the bioactive molecules from European cranberry bush fruits as reducing agents for silver ions and also as capping agents for the biosynthesized silver nanoparticles and to investigate the catalytic activity of as obtained AgNPs by testing, for the first time, their catalytic capacity in the degradation reaction by sodium borohydride of some deleterious organic colorants such as carmoisine, tartrazine and brilliant blue FCF. 

Carmoisine, also known as Acid Red 14, Azorubine S or Food Red 3 is a synthetic azo-dye, consisting in two naphtalenic subunits, widely used in foods but also in cosmetics and drugs. Tartrazine is an acidic lemon-yellow azo-dye primarily used in the food industry but with large applications in cosmetic products, pharmaceutics, inks and household cleaning products. Tartrazine appears to cause the most severe allergic reactions of all azo-dyes and therefore finding a suitable catalyst to remove this hazardous dye and to treat tartrazine contaminated wastewater is very important [32]. Brilliant blue FCF is a triarylmethane dye known also as Acid Blue 9, used for processed foods, cosmetics, dietary supplements or medications. It can induce reproductive and neurological disorders, but also allergic reactions [33], hence finding methods of removing it from water resources is necessary.

**Figure 1:**
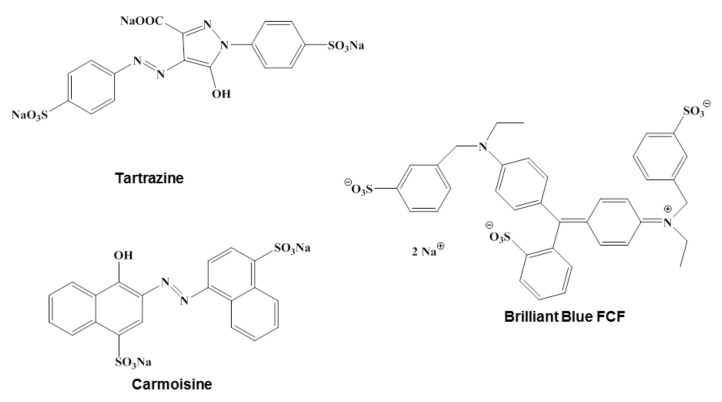
The chemical structures of investigated dyes.

The chemical structures of the investigated dyes are depicted in Figure 1**.**

## 2. Materials and Methods 

### 2.1. Reagents

Silver nitrate, sodium borohydride, sodium hydroxide, sodium carbonate, Folin–Ciocalteu reagent, carmoisine, tartrazine and brilliant blue FCF were purchased from Merck (Merck, Darmstadt, Germany) and were of analytical purity.

### 2.2. Preparation of the Fruit Extract

*Viburnum opulus* fruits were harvested in September 2019 from Cluj-Napoca, Romania. The fruits were washed three times with distilled water. An amount of 5 g of fully ripened *Viburnum opulus* fruits was ground into a fruit puree and then 100 mL of de-ionized water were added. The obtained mixture was stirred for 2 h at 600 rpm at room temperature and then filtered to get the desired extract.

### 2.3. Total Phenolic Content Evaluation

The Folin–Ciocalteu method [34] was used to assess the total phenolic content of the *Viburnum opulus* fruits extract. Samples of 0.25 mL fruit extract were mixed with 1.5 mL of Folin–Ciocalteu reagent and the mixtures were incubated for 5 min in the dark. After that, 1.2 mL of 0.7 M sodium carbonate solution was added and mixed thoroughly. The resulting solutions were allowed to react 2 h in the dark at room temperature. The obtained blue solutions were transferred into 1 cm path length quartz cuvettes and their absorbance was read at 765 nm using a Perkin-Elmer Lambda 25 UV-Vis spectrophotometer (PerkinElmer Inc., Waltham, MA, USA). The results were fitted to a calibration curve of gallic acid standard solution and the calculated total phenolic contents were expressed as mg gallic acid equivalents (GAE)/L. All measurements were done in triplicate.

### 2.4. Synthesis of Silver Nanoparticles Using Viburnum opulus Fruit Extract

The synthesis of silver nanoparticles was accomplished using a green route and exploiting the reducing capacity of phytocompounds present in *Viburnum opulus* fruit extract. To this end, 10 mL of extract were added in a drop wise manner to 50 mL aqueous silver nitrate solution (1 mM) under continuous stirring. 0.1 M aqueous NaOH solution was added to the reaction mixture in order to adjust the pH to 7.5. The rapid reduction of the silver ions to AgNPs was visually observed as the color of the reaction medium changed from faint red to yellowish brown. The obtained AgNPs suspension was subjected to repeated centrifugations at 10,000 rpm for 30 min. After removing the supernatant, the obtained pellet was further washed with double distilled water for the purification of the AgNPs. The purified pellet was dried and the characterization of the silver nanoparticles was performed by various appropriate techniques.

### 2.5. Characterization of Silver Nanoparticles 

The optical absorbance of the obtained silver nanoparticles was measured using a Perkin Elmer Lambda 25 UV-Vis spectrophotometer. The spectra were recorded within a wavelength range of 300–800 nm with a resolution of 1 nm in a quartz cuvette of 1 cm path length. The size and morphology of the bio-inspired silver nanoparticles was scrutinized by transmission electron microscopy using a H-7750 120 kV automatic TEM (Hitachi, Tokyo, Japan). TEM samples were prepared on a carbon coated copper grid by dropping AgNPs colloidal solution and drying it in vacuum. FTIR studies were carried out on a Bruker Vector 22 FT-IR spectrometer (Bruker, Dresden, Germany) in order to identify the presence of biomolecules from the extract at the surface of the phytosynthesized AgNPs. FTIR spectra of the samples were recorded in KBr pellets. The scanning was accomplished within the 4000–400 cm^−1^ range with a resolution of 4 cm^−1^. X-ray diffraction analysis of the purified dried silver nanoparticles was conducted using a D8 Advance X-ray diffractometer (Brucker, Dresden, Germany) with CuK_α_1 radiation. The observed diffraction pattern was compared to standards to account for the crystalline structure of the AgNPs. The dried powder of the biologically obtained AgNPs was subjected to thermogravimetric analysis. The TGA was accomplished using a SDT Q600 (TA Instruments, New Castle, DE, USA) analyzer under nitrogen atmosphere at a heating rate of 10°/min in a temperature range of 40–1000 °C.

### 2.6. Catalytic Activity of Silver Nanoparticles

The catalytic ability of the obtained AgNPs was assessed on three organic dyes: tartrazine, carmoisine and brilliant blue FCF in the presence of sodium borohydride. The experiments were performed by mixing 2.5 mL of dye solution (0.1 mg/mL) in de-ionized water, 0.5 mL 0.1 M sodium borohydride solution and 0.75 mL colloidal silver solution of different concentrations (20, 40 and 60 µg AgNPs/mL). The time dependent change in absorbance of the reaction mixtures was periodically monitored at room temperature at 504 nm (Cs), 420 nm (Tz) and 629 nm (BB) using UV-Vis spectrophotometry, in order to assess the residual concentration of the dye in the reaction media. The data were used to study the kinetics of the degradation reactions. Additionally, control degradation reactions were carried out without addition of the phytosynthesized silver nanoparticles. For the investigation of the reusability of the nanoparticles as a green catalyst for dye degradation, they were collected by centrifugation of the reaction mixtures, washed three times with de-ionized water and then reused in the dye reduction reaction.

## 3. Results and Discussion

### 3.1. Synthesis and Characterization of Silver Nanoparticles

*Viburnum opulus* fruits have been widely reported to possess remarkable antioxidant activity due to their high content of secondary metabolites with antioxidant potential such as anthocyanins, flavonoids, phenolic acids, tannins and vitamins [35,36]. The total phenolic content of the fruit extract was determined by the Folin–Ciocalteu method and was found to be 211.3 mg GAE/L. The high content of phenolic compounds enabled us to select these fruits as valuable candidates for the bio-inspired green synthesis of silver nanoparticles by using *Viburnum opulus* fruit extract as a source of reducing biomolecules for the silver ions and capping agents of the obtained silver nanoparticles conferring them a high stability.

**Figure 2:**
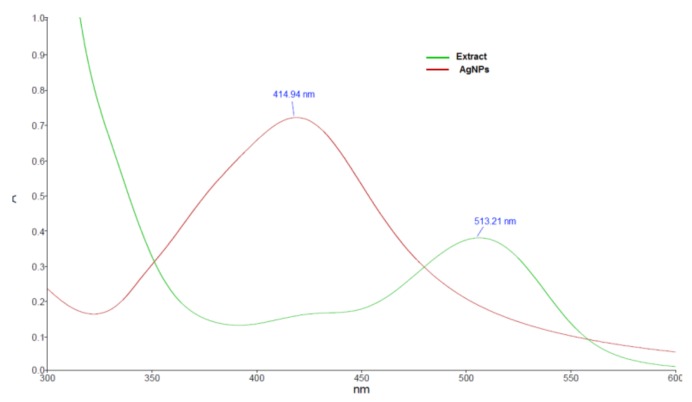
UV-Vis spectra of fruit extract and obtained silver nanoparticles.

In order to confirm the formation of the AgNPs, UV-Vis spectrophotometric analysis was used. The reduction of the silver ions was observed by recording the absorption spectrum of the silver nitrate—*Viburnum opulus* fruit extract mixture. The reduction took place very fast, as noticed also by changing of the mixture color from faint red to yellowish-brown. The UV-Vis spectra of the fruit extract and the obtained AgNPs are shown in Figure 2. The shift of the absorption maximum from 513 nm, as observed in the extract spectrum, to 415 nm indicates that the phytocompounds present in the extract successfully reduced the Ag^+^ ions into AgNPs, as it is well known that colloidal silver shows a typical absorption band in the range of 350–450 nm as a consequence of the surface plasmon resonance of the conducting electrons of the metal.

**Figure 3:**
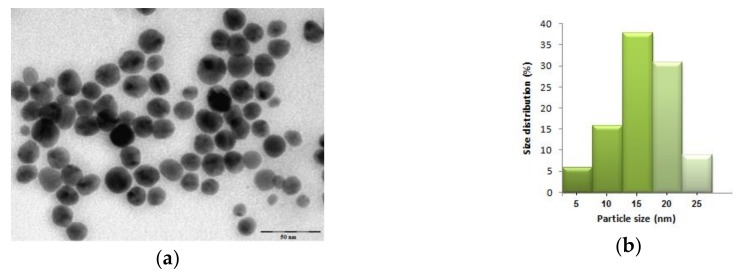
(**a**) TEM image and (**b**) histogram of obtained silver nanoparticles.

The size and morphology of the biosynthesized silver nanoparticles were characterized using TEM analysis. The TEM image (Figure 3a) revealed that the AgNPs were spherically in shape, uniformly distributed and their sizes varied between 7 and 26 nm. The particle size distribution given in Figure 3b shows a mean size of the obtained nanoparticles of 16 nm.

**Figure 4:**
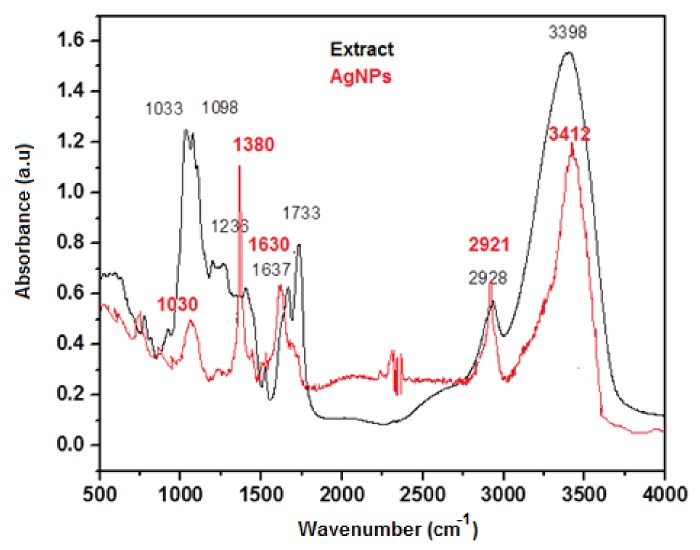
FTIR spectra of the fruit extract and phytosynthesized AgNPs.

In order to identify the organic molecules from the *Viburnum opulus* fruit extract that acted as reducing agents of the silver ions and capping the obtained nanoparticles, Fourier transformed infrared spectroscopy was applied. The FTIR spectra of the fruit extract and phytosynthesized AgNPs are shown in Figure 4. The spectra revealed the presence of different functional groups in the biomolecules and their possible involvement in the synthesis and stabilization of the silver nanoparticles. Both spectra presented similar IR absorption bands, confirming the presence of organic compounds from the fruit extract at the surface of the silver nanoparticles limiting their agglomeration. The absorption bands were slightly shifted in the IR spectrum of the AgNPs as compared to those that appeared in the fruit extract spectrum. In the case of crude extract, the broad band around 3398 cm^−1^ indicates the stretching vibration of the OH groups of the polyphenols present in the fruit extract, the band that was slightly shifted at 3398 cm^−1^ in the IR spectrum of AgNPs. The C=O stretching at 1733 cm^−1^ in the extract spectrum disappeared. The bands observed at 2921, 1380 and 1030 cm^−1^ were attributed to C–H stretching vibration, C–O stretching vibration and C–O bending frequencies, respectively. All these bands confirmed that the main plant metabolites from the fruit extract involved in the synthesis of AgNPs are polyphenols, phenolic acids and flavonoids, results in agreement with previous studies [12,37].

**Figure 5:**
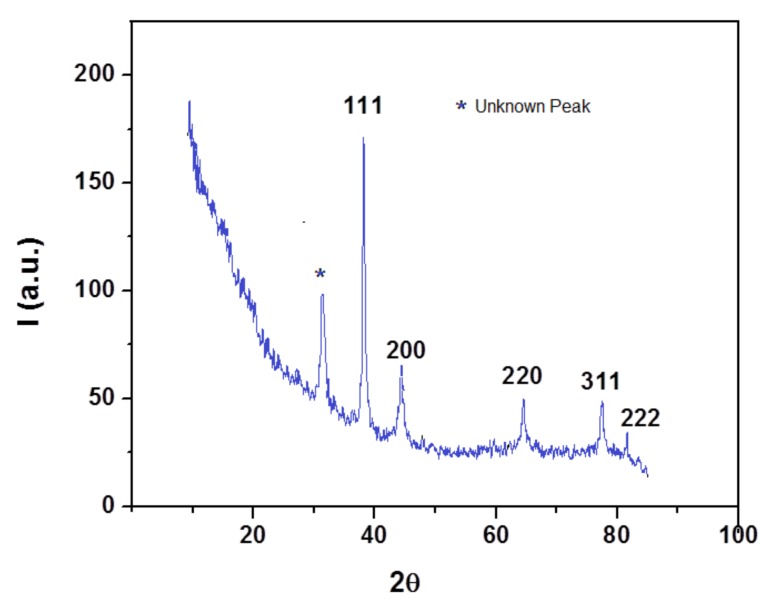
XRD pattern of the AgNPs.

The X-ray diffraction analysis (XRD) was used to investigate the crystallinity of the nanoparticles. The powder XRD pattern of the AgNPs is shown in Figure 5. The diffraction peaks, corresponding to (111), (200), (220), (311) and (222) of the face centered cubic structure of metallic silver, were observed at 38.33°, 44.56°, 64.62°, 77.44° and 82.41°, in accordance to the reference of the FCC structure of the Joint Committee of Powder Diffraction Standard (JCPDS file no. 04−0783). The XRD profile also exhibited the presence of an additional peak, appearing at 2θ value of 32.2°, which might be due to the presence of organic compounds in the sample, results in agreement with those reported in previous studies [38].

**Figure 6:**
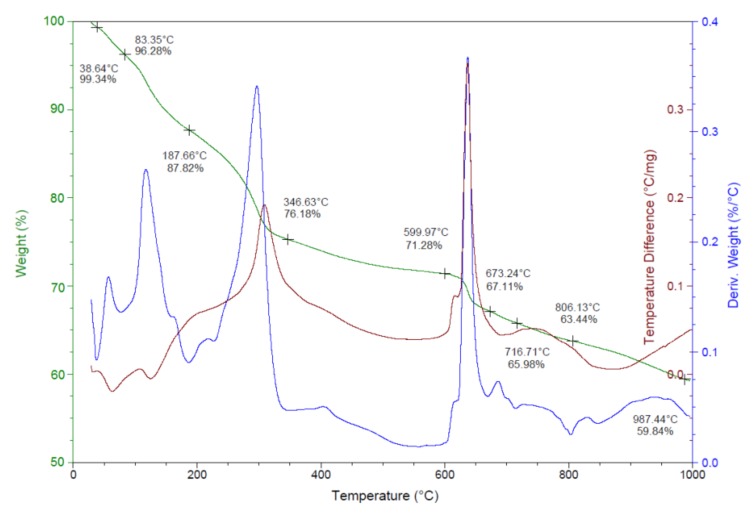
TGA/DSC/DTG graphs of silver nanoparticles.

The thermal behavior of the obtained silver nanoparticles was examined by thermogravimetric analysis–differential scanning calorimetry (DSC) and derivative thermogravimetry (DTG). The TGA/DSC/DTG graphs of the phytosynthesized AgNPs are shown in Figure 6.

The first step of the decomposition process, which occurred between 40 and 100 °C, might be attributed to the evaporation of moisture adsorbed on the surface of the AgNPs. A further degradation, with an associated weight loss of about 19%, was observed between 100 and 350 °C, which is a consequence of the desorption of the organic biomolecules present at the surface of the nanoparticles. This weight loss might be attributed to the decomposition of phenolic acids, flavonoids and carbohydrates, which originate in the fruit extract and are responsible for the stabilization of AgNPs [12]. Above 350 °C, a steady weight loss accounting about 16% was recorded, which could probably be assigned to the thermal degradation of resistant aromatic compounds present on the surface of the silver nanoparticles [39]. The TGA data clearly indicated that the bioactive organic compounds from the fruit extract are attached to the surface of the obtained nanoparticles and the organic shell was found to be 35.14%, results in agreement with those reported by other studies [39,40]. The DSC curve indicates the presence of endothermic and exothermic processes. The endothermic peaks at 38.64 °C and 83.35 °C were due to the elimination of water molecules while the three exothermic peaks at 125 °C, 300 °C and 640 °C correspond to the desorption of the organic components bound to the surface of the silver nanoparticles [41].

### 3.2. Dye Reducing Catalytic Activity of AgNPs

The catalytic capacity of the silver nanoparticles obtained with *Viburnum opulus* fruit extract was evaluated in the reduction reaction of three organic dyes: carmoisine, tartrazine and brilliant blue FCF.

**Figure 7:**
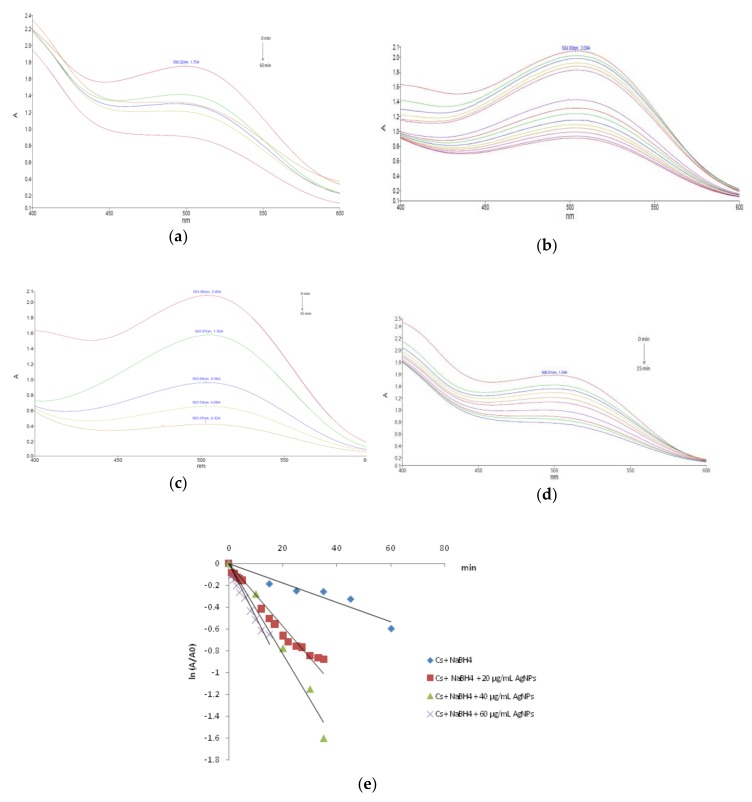
NaBH_4_ mediated carmoisine degradation: (**a**) without catalyst; (**b**) in the presence of 20 µg/mL AgNPs; (**c**) in the presence of 40 µg/mL AgNPs; (**d**) in the presence of 60 µg/mL AgNPs and (**e**) kinetics plots.

The sodium borohydride mediated reduction of carmoisine in the absence of silver nanoparticles occurred slowly. After 60 min, as shown in Figure 7a, only 45% of carmoisine was reduced, as calculated from the intensity of the absorption peak at 504 nm. However, the addition of catalytic amounts of synthesized AgNPs in the reaction mixture significantly accelerated the degradation reaction. This was easily noticed from the fading of the red color as well as the decrease in the intensity of the absorption peak at 504 nm. The influence of the catalyst concentration on the degradation process of carmoisine was investigated. Experiments were carried out by using different amounts of AgNPs for the degradation of the dye molecules, by keeping constant the dye concentration. The reaction mixtures in the presence of the various amounts of the AgNPs catalyst was spectrophotometrically monitored at different time intervals over a period of 15–60 min, related to the degradation rate of the carmoisine dye. Figure 7 illustrates the UV-Vis spectra recorded during the dye degradation in the presence of 20 µg/mL AgNPs (Figure 7b), 40 µg/mL AgNPs (Figure 7c) and 60 µg/mL AgNPs (Figure 7d). The increase of the reaction rate by adding the phytosynthesized silver nanoparticles to the mixture of carmoisine and sodium borohydride is mainly due to the silver nanoparticles mediated electron transfer. The electron transfer plays a fundamental role in the degradation of the organic azo dyes. The reduction of carmoisine by sodium borohydride in the absence of AgNPs is thermodynamically but not kinetically favorable, due to the large difference in redox potential between the donor and the acceptor. The silver nanocatalyst is able to reduce the kinetic barrier by providing an alternative route of low activation energy for the reduction reaction, making it also kinetically favorable [42,43].

The kinetic parameters of the borohydride mediated carmoisine degradation in the presence of silver nanoparticles as a catalyst were determined by monitoring the change in the dye concentration with respect to the reaction time. As the concentration of sodium borohydride was adjusted to a much higher level as the carmoisine concentration, it remains practically unchanged during the degradation reaction. As a consequence, the kinetics of reduction reaction of the Cs in the investigated conditions can be regarded as pseudo-first order kinetics with respect to carmoisine. Thus, the reaction kinetics can be described by the pseudo-first order rate law, by using the equation:

ln (A/A_0_) = −k × t, (1)

where A_0_ = absorbance of Cs at time t = 0, A = absorbance of Cs at time t, k = reaction rate constant and t = reaction time. The linear regression of ln (A/A_0_) versus time confirmed the pseudo-first order nature of the Cs reduction reaction for all the investigated concentrations of the nanocatalyst (Figure 7e). The slope of the obtained plots was used to calculate the pseudo-first order rate constants. The kinetic parameters and the corresponding correlation coefficients are given in Table 1. The obtained data clearly indicate the catalytic effect of the phytosynthesized silver nanoparticles as the carmoisine reduction reaction occurred three to five times faster in the presence of the nanocatalyst. Regarding the influence of the catalyst concentration, the dye degradation rate increased with increasing amount of the AgNPs. The most effective degradation of carmoisine was observed when the catalyst amount was 60 µg/mL. This could be attributed to the increase of the catalyst surface available for the absorption of the reactants.

**Figure 8:**
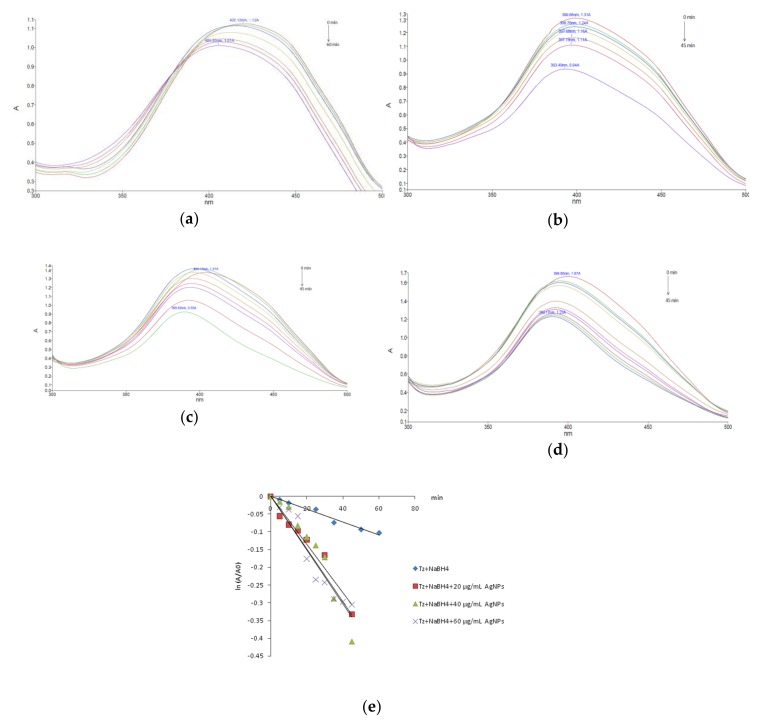
NaBH_4_ mediated tartrazine degradation: (**a**) without a catalyst; (**b**) in the presence of 20 µg/mL AgNPs; (**c**) in the presence of 40 µg/mL AgNPs; (**d**) in the presence of 60 µg/mL AgNPs and (**e**) kinetics plots.

The catalytic performance of the *Viburnum opulus* mediated phytosynthesized silver nanoparticles was also evaluated on the reduction reaction of tartrazine in the presence of the same reduction agent (NaBH_4_). The UV-Vis spectra of the tartrazine borohydride mixture in the absence and after adding different amounts of the nanocatalyst exhibited a slow or accelerated reduction of the absorption band at 420 nm as shown in Figure 8.

As can be observed, the concentration of tartrazine slowly decreased in the absence of silver nanoparticles, only 28% of the total amount of dye being degraded after 1 h. By adding silver nanoparticles, as the catalyst of the reduction reaction, the larger surface area provided by the nanocatalyst led to an increase of the degradation rate. The investigation of the reaction kinetics, by plotting ln (A/A_0_) versus reaction time indicated, as in the case of carmoisine, a pseudo-first order reaction kinetics (Figure 8e). The rate constants were obtained from the slope of the linear kinetic plots and where further used to estimate the half-life t_1/2_ of each degradation process, according to the equation:

t_1/2_ = −ln0.5/k, (2)

where t_1/2_ = half-life value and k = rate constant.

The presence of the nanocatalyst accelerated the reaction, by increasing the degradation rate of about four folds. The obtained values are given in Table 1.

**Figure 9:**
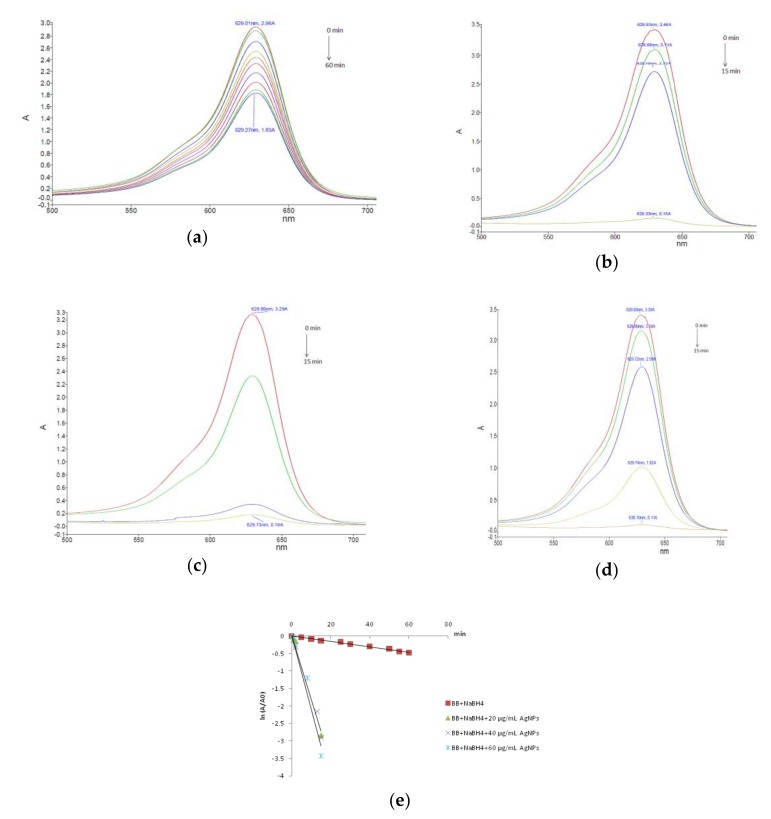
NaBH_4_ mediated brilliant blue FCF degradation: (**a**) without a catalyst; (**b**) in the presence of 20 µg/mL AgNPs; (**c**) in the presence of 40 µg/mL AgNPs; (**d**) in the presence of 60 µg/mL AgNPs and (**e**) kinetics plots.

The reductive degradation of brilliant blue FCF in the presence of sodium borohydride occurred relatively slow, after 1 h of reaction, only 38% of the dye being degraded (Figure 9a). Adding the green synthesized silver nanocatalyst, the degradation reaction was highly accelerated as observed in the UV-Vis spectra of the reaction mixtures presented in Figure 9. The brilliant blue FCF reduction reaction also followed a pseudo-first order kinetic model (Figure 9e). The catalytic efficacy of AgNPs related to this triarylmethane dye was by far the most potent of all the investigated dyes. The kinetic parameters, given in Table 1, revealed a large increase of the rate constant in the presence of the nanocatalyst, the reaction running 27 times faster when 60 µg/mL AgNPs were added to the borohydride dye mixture.

Our results confirmed the efficacy of silver nanoparticles as a valuable catalyst for toxic organic dyes degradation, results also obtained by other researchers. The green synthesized AgNPs were successfully used for the degradation of hazardous dyes such as methylene blue, methyl orange, direct yellow 12, reactive black 5, rhodamine B, Congo red and so on. All the tested dyes underwent rapid degradation in the presence of a silver nanocatalyst, following pseudo-first order reaction kinetics [26,44,45]. Silver nanoparticles mediated catalytic reductive degradation of azo dyes has been largely investigated due to its efficacy. Edison et al. [31] successfully employed *Anacardium occidentale* testa derived AgNPs to degrade carcinogenic azo dyes such as Congo red and methyl orange, in the presence of NaBH_4_, reactions that occurred with a high rate, obtaining a rate constant of 0.0795 and 0.1178 min^−1^ for the degradation of Congo red and methyl orange, respectively, rate constants, which are similar to those obtained in this work by using *Viburnum opulus* mediated AgNPs (Table 1). The catalytic role of green synthesized silver nanoparticles in the degradation of organic dyes was also confirmed by Josef and Mathew [29], which used *Biophytum sensitivium* leaf extract as a green agent for the synthesis of AgNPs. The obtained nanoparticles proved to be efficient catalysts in the NaBH_4_ mediated degradation of methyl orange, a reaction that also occurred by pseudo-first order kinetics with rate constants from 0.1953 to 0.2758 min^−1^ depending on AgNPs concentration. All these results clearly confirmed that plant mediated synthesized silver nanoparticles are efficient catalysts for the reductive degradation of harmful organic dyes.

Despite the numerous efforts conducted in finding the most efficient way to degrade the harmful organic dyes, phytosynthesized AgNPs have not been yet employed as catalysts of Tartrazine, Carmoisine or Brilliant Blue FCF degradation. Compared to other techniques/nanomaterials, the reductive degradation of these dyes in the presence of our biogenic AgNPs proved to be an efficient way to remove these toxic pollutants. The photocatalytic degradation of Acid red 14 (Carmoisine) using immobilized TiO_2_ nanoparticles on graphene oxide proved to be an efficient degradation method, resulting in a 69.9% discoloration of dye after 120 min, as reported by Akerdi et al. [46]. Compared to this process, the study by us investigated the degradation of Cs in the presence of AgNPs, occurred faster, after 15 min 48% of the dye had been degraded.

**Figure 10:**
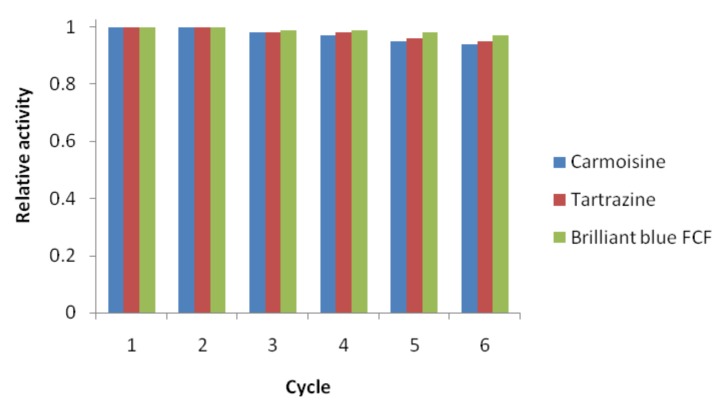
Reusability of silver nanoparticles for the reduction of organic dyes with NaBH_4._

Scott et al. reported the oxidative degradation under UV light of Tz and BB, which resulted in a slow process. After 300 min of UV light exposure, 83% of BB and only 17% of Tz underwent degradation [47]. Taking into account that using silver nanoparticles as catalysts of the degradation reactions of the above mentioned dyes, Tz concentration lowered by 26% after 45 min of reaction and 97% of BB was removed after 15 min, is highly recommended to use the catalytic degradation of these dyes instead of their UV light mediated oxidation. The photocatalytic degradation of BB was also investigated in the presence of in situ modified tungsten doped TiO_2_ hybrid nanoparticles [48], but this nanocatalyst also proved to be less efficient compared to *Viburnum opulus* mediated synthesized AgNPs. The determined degradation rate constant obtained in the presence of TiO_2_ nanoparticles was 0.075 min^−1^, while the presence of the biogenic silver nanocatalyst accelerated the degradation of BB, the calculate rate constant being almost three folds higher (0.2097 min^−1^). Yousefi et al. investigated the oxidative degradation of Brilliant Blue FCF (BB) in the presence of zero valent iron as a catalyst and the degradation took place in 30 min [33], while using the green synthesized AgNPs as a catalyst for BB degradation, the reaction was twice faster (15 min). Using modified manganese-added ZnO nanoparticls as photocatalysts of BB degradation, Shahmoradi et al. [49] obtained a photodegradation of cca. 85% of the dye after 3 h, results proving that the silver nanocatalyst was also, by far, more efficient than the ZnO nanoparticles. The reusability of the nanocatalyst in the dye degradation reduction reactions is a very important parameter for their exploitation as efficient catalysts. In order to investigate this parameter, the AgNPs were recovered from the reaction mixtures by centrifugation, thoroughly washed to remove the attached undesired species from their surface and reused in the NaBH_4_ reduction of the organic dyes. The degradation rate of all the colorants presented no significant decrease for the first three cycles, but after the third cycle, a slight activity decrease was observed in the reuse of the nanocatalyst, as shown in Figure 10, attributed to the deactivation of the reaction sites on the catalyst surface. The results suggest that the phytosynthesized silver nanoparticles obtained in this work were relatively stable and could be successfully used as a efficient catalyst with a slight decrease in the degradation rate for more than five times.

## 4. Conclusions

The present study reported a green, simple, one pot synthesis method for obtaining silver nanoparticles by using the bioactive components from the *Viburnum opulus* fruit extract as reducing and stabilizing agents. The obtained nanoparticles were characterized using UV-Vis, TEM, XRD, FTIR and TGA analysis. The results confirmed the production of well dispersed, spherical, uniform sized AgNPs, having an average diameter of 16 nm. The catalytic ability of the AgNPs was investigated on the degradation reaction of three hazardous organic dyes: carmoisine, tartrazine and brilliant blue FCF, proving a high efficacy for all the investigated colorants, presenting an outstanding catalytic activity against brilliant blue FCF degradation. As a consequence of these results, the *Viburnum opulus* mediated synthesized nanoparticles could be efficiently used in the field of environmental remediation and their remarkable activity could be successfully exploited to degrade the harmful organic dyes from various industrial effluents.

## Figures and Tables

**Table 1:** Kinetic parameters of degradation reactions of organic dyes.

Colorant/Catalyst Concentration	k (min^−1^)	t_1/2_ (min)	R^2^
**Brilliant Blue**			
No catalyst	0.0077	90.00	0.988
20 µg/mL AgNPs	0.1817	3.81	0.987
40 µg/mL AgNPs	0.1864	3.71	0.987
60 µg/mL AgNPs	0.2097	3.30	0.958
**Tartrazine**			
No catalyst	0.0018	385	0.978
20 µg/mL AgNPs	0.0068	101.92	0.956
40 µg/mL AgNPs	0.0071	93.6	0.891
60 µg/mL AgNPs	0.0076	91.18	0.929
**Carmoisine**			
No catalyst	0.0090	77	0.916
20 µg/mL AgNPs	0.0287	24.14	0.961
40 µg/mL AgNPs	0.0416	16.81	0.969
60 µg/mL AgNPs	0.0496	13.97	0.943
